# Healthy lifestyle and genomic ancestry related to good glycemic control in type 1 diabetes patients from Northeastern Brazil: a hierarchical analysis

**DOI:** 10.3389/fendo.2023.1233050

**Published:** 2023-10-13

**Authors:** Rossana Santiago de Sousa Azulay, Vandilson Rodrigues, Joana D’Arc Matos França de Abreu, Ana Gregória Ferreira Pereira de Almeida, Débora Lago, Maria da Glória Tavares, Gilvan Nascimento, Viviane Rocha, Marcelo Magalhães, Alexandre Facundo, Clariano Pires de Oliveira Neto, Adriana Guimarães Sá, Conceição Ribeiro Veiga Parente, Dayse Aparecida Silva, Marília Brito Gomes, Manuel dos Santos Faria

**Affiliations:** ^1^ Service of Endocrinology, University Hospital of the Federal University of Maranhão (HUUFMA), São Luís, Brazil; ^2^ Research Group in Clinical and Molecular Endocrinology and Metabology (ENDOCLIM), Federal University of Maranhão, São Luís, Brazil; ^3^ DNA Diagnostic Laboratory (LDD), Rio de Janeiro State University (UERJ), Rio de Janeiro, Brazil; ^4^ Diabetes Unit, Rio de Janeiro State University (UERJ), Rio de Janeiro, Brazil

**Keywords:** type 1 diabetes, medical nutritional therapy, physical activity, genetic profile, glycated hemoglobin

## Abstract

**Introduction:**

This study aimed to investigate the sociodemographic factors, dietary adherence, regular physical activity, and genomic ancestry percentage associated with good glycemic control in Brazilian patients with type 1 diabetes (T1D) using a hierarchical approach.

**Methods:**

A cross-sectional study was conducted in 152 T1D patients. Glycated hemoglobin (HbA1C) levels were measured to evaluate the glycemic control status (good, moderate, or poor). Independent factors included sex, age, self-reported skin color, educational level, family income, dietary patterns, and physical activity. The percentage of genomic ancestry (Native American, European, and African) was influenced by a panel of 46 autosomal insertion/deletion ancestry markers. Statistical analyses included receiver operating characteristic curves, and hierarchical logistic regression analysis.

**Results:**

The hierarchical analysis, patients who had high dietary adherence showed a positive association with good glycemic control (_adjusted_OR = 2.56, 95% CI:1.18-5.59, P = 0.016). Thus, age greater than 40 years was associated with good glycemic control compared to the children and adolescents group (_adjusted_OR = 4.55, 95% CI:1.14-18.1, P = 0.031). Males were associated with good glycemic control (_adjusted_OR = 2.00, 95% CI:1.01-4.00, P =0.047).

**Conclusion:**

The study findings suggest that consistent adherence to dietary regimens is associated with good glycemic control after adjusting for sociodemographic and genomic ancestry factors in an admixed population of T1D patients from Northeast Brazil.

## 1 Introduction

Type 1 diabetes (T1D) is characterized by hyperglycemia, which results from an insulin deficit caused by the autoimmune destruction of pancreatic beta cells. Thus, it is understandable that the mainstay of the treatment of this pathology is exogenous insulin replacement (1, 2). However, to achieve good metabolic control, two other pillars of diabetes treatment are necessary: diet and exercise (3, 4). The adherence to these healthy lifestyle behaviors as well as access to health care services could effectively prevent the risk of complications associated with poor glycemic control.

The guidelines of important international (4, 5) and Brazilian (6) societies that guide the treatment of T1D, suggest that medical nutritional therapy (MNT) should be individualized with respect to cultural, family, financial, and self-management characteristics, including general guidelines for healthy eating and adequacy of the number of carbohydrates. In a systematic review and meta-analysis, carbohydrate counting (CHOC) was found to be superior in reducing glycated hemoglobin (HbA1c) levels compared with the usual diabetes diet (7, 8). It is important to highlight that low adherence to nutritional therapy is associated with poor glycemic control (9, 10).

Pieces of evidence have shown the beneficial effects of physical activity practice on weight control, reducing cardiovascular risk, improving quality of life (11, 12), and reducing neuroticism (13, 14). A recent review article indicated that physical activity is a useful non-pharmacological tool for improving glycemic control (15), although the benefits of glycemic control do not have a robust evidence base in T1D patients (11, 12). These diverse findings may be explained by the differences in methodology between studies. Another important positive aspect is that physical activity, especially when performed regularly and with moderate intensity, is related to improve the well-being and psychological health of children and adolescents with T1D (12).

Glycemic control can be assessed using several methods; however, HbA1c is the main measure used in studies and is adequately correlated with the risk of diabetic complications (16). Maintaining good glycemic control is associated with a lower incidence of chronic complications of diabetes (17, 18), and is the main treatment for these patients. However, this is difficult for most patients (19–21), particularly Hispanic white (22) and black individuals (22, 23).

Healthy lifestyle behaviors can have different effect on glycemic control in distinct genetic backgrounds. Therefore, this study aimed to analyze the influence of adherence to diet and regular exercise on the acquisition of good glycemic control in patients with T1D, and whether this influence is independent of the percentage of genomic ancestry in a highly admixed population in Northeast Brazil.

## 2 Materials and methods

### 2.1 Study design and samples

We present data from a cross-sectional study conducted in São Luís, Maranhão, Brazil. This research was approved by the ethics committee from the University Hospital of the Federal University of Maranhão (HUUFMA) under opinion number 59795116.9.0000.5086, in accordance with the current regulations. Participants or their legal representatives signed an informed consent form.

Patients aged >10 years old with a diagnosis of T1D according to classic clinical criteria (polyuria, polydipsia, polyphagia and weight loss) associated with insulin therapy since diagnosis were included. Exclusion criteria were as follows: patients with a history of acute infectious disease or diabetic ketoacidosis in the 3 months prior to data collection, pregnancy and lactation.

### 2.1 Data collection

Patients with T1D submitted to a clinical-demographic inquiry through a standardized questionnaire in which data related to sex, age (years), body mass index (BMI kg/m²), self-reported color/race based on the classification of the IBGE (Brazilian Institute of Geography and Statistics): black, white, brown and indigenous  (24), family income (number of BMW = Brazilian minimum wage), age at diagnosis (years), duration of T1D (years), diet (MNT-characteristics and adherence) and level of physical activity. The patients received nutritional and physical activity orientation appropriate to their medical, intellectual, and social conditions, being, therefore, quite diversified. Participants were asked about the type of diet they followed (only sugar restriction, carbohydrate counting, or personalized dietary guidance) and difficulties in following the prescribed diet (avoiding sugar and sweet foods, eating vegetables and fruits, the quantity of prescribed foods, meal times, and understanding food replacement lists). Adherence to the diet was considered good when the patient reported following the proposed diet at least 80% of the time and regular physical activity if performed three or more times a week (25–27).

### 2.2 Laboratory analysis

Peripheral blood collection was performed to measure glycated hemoglobin A1c (HbA1c) (by HPLC; reference values:4.0-6.0%) and we considered “good glycemic control” when HbA1c was lower than 7% in adults (over 19 years old) (16) and less than 7.5% in children and adolescents (due to conditions complicating control in most of our pediatric population) (4). HbA1c levels greater than 9% were considered indicative of “poor glycemic control”.

We used an SP QIA Symphony commercial kit (Qiagen, Germantown, MD, USA) according to the manufacturer’s instructions to guarantee DNA in peripheral blood samples.

To analyze genomic ancestry, a panel of 46 informational markers of autosomal insertion/deletion ancestry (AIM-indels) was amplified in a single multiplex PCR according to the protocol described by Pereira et al. (28). Polymorphisms in the generated fragments were identified by capillary electrophoresis, using an ABI 3500 automatic sequencer (Life Technologies). Genotyping was performed using the GeneMapper Analysis Software v.4.1 (Life Technologies). To predict ancestry, Structure v.2.3.3 software was used.

### 2.3 Statistical analysis

Data analysis was performed using R software (http://www.R-project.org) and GraphPad Prism version 9 (GraphPad Software Inc., San Diego, USA). Data are presented as frequencies, means, and standard deviations. The normality of continuous data was checked with the Shapiro-Wilk test and homogeneity with the Levene test. The independent t-test, Mann-Whitney U test, and one-way ANOVA were used to compare HbA1c levels. Receiver operating characteristic (ROC) curves were used to estimate the area under the curve (AUC) and 95% confidence intervals (95% CI) for Native American, European, and African ancestry percentages to predict good glycemic control.

For categorical variables, the chi-squared test and odds ratio (OR) were used to test the association with good glycemic control. Furthermore, hierarchical logistic regression was conducted using three levels (Sociodemographic, Genomic ancestry, and lifestyle data) to determine their association with good glycemic control. Model 1 was adjusted for age, sex, self-reported skin color, education, and family income. Model 2 was adjusted for Native American, European, and African ancestry in addition to Model 1 covariates. Model 3 was adjusted for dietary adherence, regular physical activity, and Models 1 and 2 covariates. The level of significance was set at P <0.05.

## 3 Results

A total of 152 T1D patients (73 females and 79 males) were included in this study. The sociodemographic distribution showed a higher percentage of the brown skin color group and a family income of more than 1 BMW in the study sample. Good glycemic control was identified in only18.4% of the sample (**Table 1**).

**Table 1:** Distribution of sociodemographic and health data of the patients with T1D.

Variables	mean	± sd	n	(%)
Age (years)	25.1	± 10.6		
Sex				
Female			73	(48.0)
Male			79	(52.0)
Self-reported skin-color				
White			44	(28.9)
Brown			99	(65.1)
Black			9	(6.0)
Family income				
Up to 1 BMW			46	(30.3)
More than 1 BMW			106	(69.7)
BMI (kg/m²)	22.2	± 3.7		
Diabetes data				
Age at diabetes diagnosis (years)	11.3	± 8.1		
Time since diabetes diagnosis (years)	13.8	± 8.7		
HbA1c level (%)	9.0	± 2.3		
Glycemic control^1^				
Good			28	(18.4)
Moderate			59	(38.8)
Poor			65	(42.8)

± sd, standard deviation; BMW, Brazilian minimum wage; BMI, body mass index. ^1^ Good glycemic control was defined as HbA1c levels lower than 7% in adults (over 19 years old) and lower than 7.5% in children and adolescents. Poor glycemic control was defined as HbA1c levels greater than 9%.

Regular physical activity was reported by 30.3% of patients, while 39.5% had dietary adherence. The most frequent dietary type was personalized advice (64.8%). The most reported difficulties in following the prescribed diet were the quantity of prescribed foods (65.1%) and the schedule of meals (50%). The European ancestry mean was the highest in the sample (**Table 2**).

**Table 2:** Distribution of regular physical activity, diet pattern, and genomic ancestry percentage of the patients with T1D.

Variables	mean	± sd	n	(%)
Regular physical activity				
Yes (3 or more days/week)			46	(30.3)
No (<3 days/week)			106	(69.7)
Dietary type				
Sugar restriction			28	(19.3)
Carbohydrate counting			23	(15.9)
Personalized dietary advice			94	(64.8)
Dietary adherence				
Yes (≥80%)			60	(39.5)
No (<80%)			92	(60.5)
Difficulties to follow the prescribed diet				
Avoiding sugar and sweets			61	(40.1)
Eating vegetables and fruits			27	(17.8)
Quantity of prescribed foods			99	(65.1)
Schedule time of the meals			76	(50.0)
Understanding foods substitution lists			36	(23.7)
Global ancestry percentage				
Native American	24.6	± 9.5		
European	46.5	± 14.4		
African	28.5	± 12.7		
Native American ancestry				
Up to 40%			119	(78.3)
More than 40% up to 60%			33	(21.7)
European ancestry				
Up to 40%			37	(24.3)
More than 40% up to 60%			44	(28.9)
More than 60%			71	(46.7)
African ancestry				
Up to 40%			106	(69.7)
More than 40% up to 60%			34	(22.4)
More than 60%			12	(7.9)

±sd, standard deviation.

A comparative analysis of HbA1c levels and lifestyle data associated with good glycemic control is presented in **Table 3**. Patients who had dietary adherence had a lower HbA1c level (8.37 ± 2.19 versus 9.38 ± 8.37, P = 0.003). A higher HbA1c was identified in patients who reported difficulties in avoiding sugar and sweets (9.48 ± 1.99 versus 8.65 ± 2.33, P = 0.028). The crude analysis showed that dietary adherence was associated to increase odds of good glycemic control (OR = 2.16, 95% CI:1.09-4.26, P = 0.027), and difficulties in avoiding sugar and sweets were associated to reduce of good glycemic control (OR = 0.51, 95% CI:0.26-0.94, P = 0.049).

**Table 3:** Comparative analysis of HbA1c level according to regular physical activity and diet pattern in T1D patients.

Variables	HbA1c level			Good glycemic control		
	mean	± sd	P	OR	95% CI	P
Regular physical activity			0.157			
Yes	8.58	± 1.85		1.85	0.89–3.81	0.098
No	9.15	± 2.45		Ref.		
Dietary type			0.732			
Sugar restriction	9.16	± 2.51		Ref.		
Carbohydrate counting	8.24	± 2.46		0.81	0.27–2.48	0.723
Personalized dietary advice	8.76	± 8.76		1.26	0.53–2.98	0.592
Dietary adherence			0.003*			
Yes (≥80%)	8.37	± 2.19		2.16	1.09–4.26	0.027*
No (<80%)	9.38	± 2.26		Ref.		
Difficulties to follow the diet						
Avoiding sugar and sweets			0.028*			
Yes	9.48	± 1.99		0.51	0.26–0.94	0.049*
No	8.65	± 2.33		Ref.		
Eating vegetables and fruits			0.285			
Yes	8.56	± 2.32		1.11	0.47–2.58	0.815
No	9.07	± 2.21		Ref.		
Quantity of prescribed foods			0.589			
Yes	9.06	± 1.94		0.82	0.41–1.62	0.567
No	8.85	± 2.57		Ref.		
Schedule time of the meals			0.254			
Yes	8.77	± 2.11		1.31	0.68–2.49	0.413
No	9.19	± 2.34		Ref.		
Understanding foods substitution			0.865			
Yes	9.04	± 2.11		1.06	0.49–2.26	0.879
No	8.96	± 2.34		Ref.		

±sd, standard deviation; OR, Odds ratio; 95% CI, 95% confidence interval.

**Figure 1:**
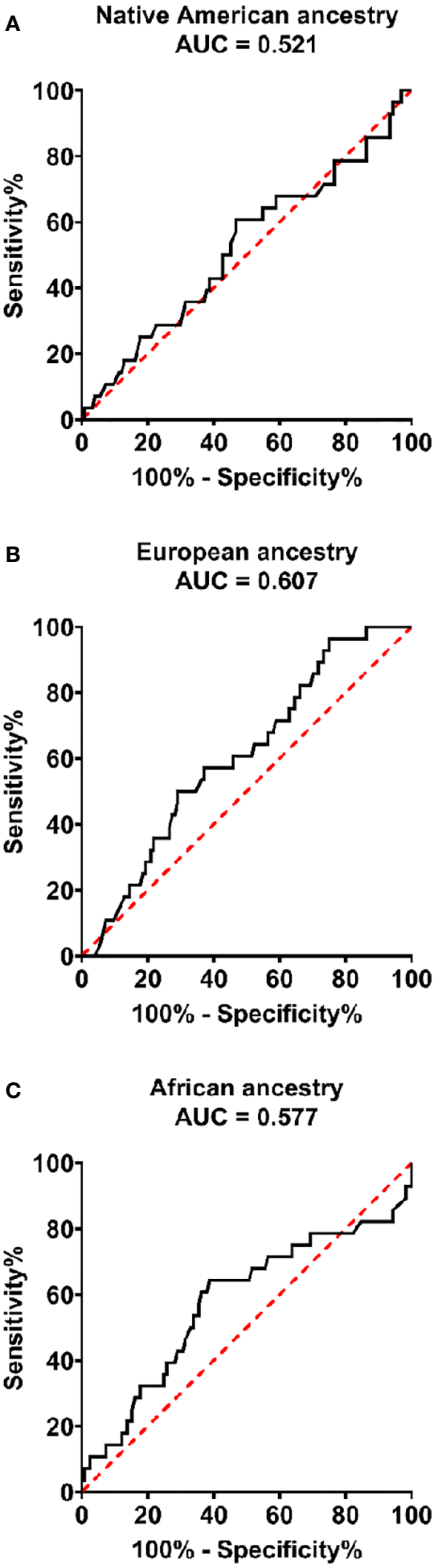
Receiver operator curves for Native American **(A)**, European **(B)**, and African **(C)** ancestry percentages to predict good glycemic control in patients with type 1 diabetes. Native American (AUC = 0.521, 95% CI:0.398-0.645, P = 0.725), European (AUC = 0.601., 0.500-0.714, P = 0.079). and African (AUC = 0.577, 95% CI:0.448–0.706, P = 0.204).

Receiver operating characteristic curves for Native, European, and African ancestry showed no statistically significant accuracy in predicting good glycemic control in the sample (**Figure 1**).

**Figure 2:**
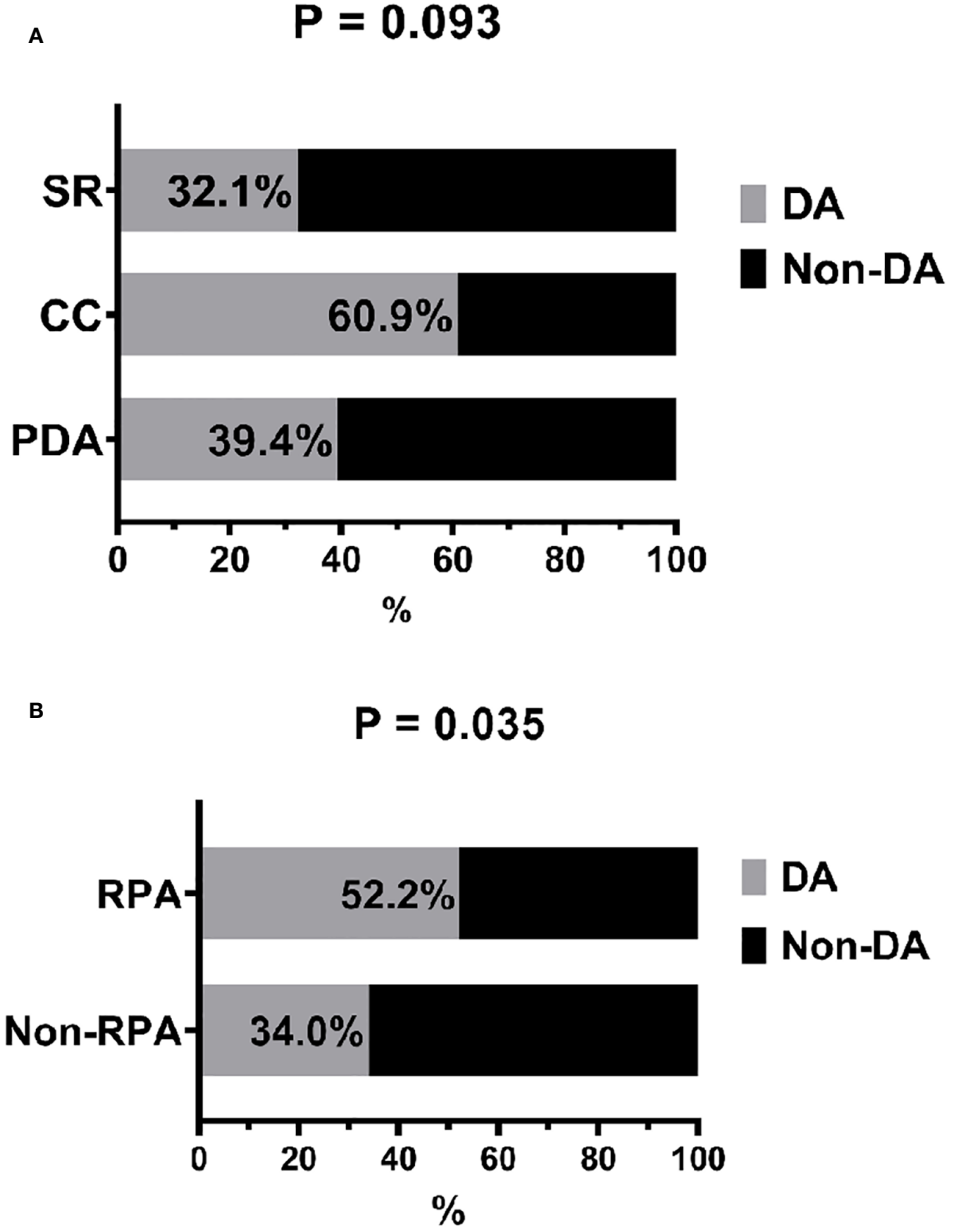
Distribution of dietary type according to dietary adherence **(A)** and frequency of dietary adherence according to regular physical activity **(B)**. DA, dietary adherence; RPA, regular physical activity; SR, sugar restriction; CC, carbohydrate counting; PDA, personalized dietary advice.

**Figure 2** shows that there was a statistically higher percentage of patients who adhered to the diet among those in the group with regular physical activity (52.2% versus 34%, P = 0.034).

The hierarchical logistic regression analysis is presented in **Table 4**. Model 1 showed that age older than 40 years was associated with good glycemic control compared to the adolescent group (_adjusted_OR = 4.55, 95% CI:1.14-18.1, P = 0.031), and males were associated with good glycemic control (_adjusted_OR = 2.00, 95% CI:1.01-4.00, P =0.047). Model 3 showed that patients who had high dietary adherence was positively associated with good glycemic control (_adjusted_OR = 2.56, 95% CI:1.18-5.59, P = 0.016).

**Table 4:** Multiple logistic regression analysis of sociodemographic, genomic ancestry and lifestyle factors associated with glycemic control.

Factors	Outcome: Good glycemic control		
	Adjusted OR	95%CI	P
Sociodemographic level (Model 1)			
Male (Ref.: Female)	2.00	1.01-4.00	0.047*
Age (Ref.: Up to 18 years)			
19 up to 40 years	1.58	0.76-3.18	0.222
≥41 years	4.55	1.14-18.10	0.031*
Self-reported skin-color (Ref.: White)			
Brown	2.35	0.41-13.44	0.338
Black	0.73	0.34-1.57	0.422
Education level (Ref.: ≤8 years)			
>8 years of education	1.37	0.53-3.50	0.506
Family income (Ref.: ≤1 BMW)			
>1 BMW	1.32	0.58-2.97	0.499
Genomic ancestry level (Model 2) (Ref.: Up to 40%)			
Native American ancestry			
More than 40% up to 60%	1.53	0.61-3.83	0.358
European ancestry			
More than 40% up to 60%	1.03	0.38-2.75	0.952
More than 60%	2.52	0.90-7.07	0.078
African ancestry			
More than 40% up to 60%	2.65	0.98-7.13	0.053
More than 60%	4.50	0.89-22.73	0.068
Healthy lifestyle level (Model 3)			
Regular physical activity			
Yes (Ref.: No)	1.32	0.58-2.99	0.506
Dietary adherence			
Yes (Ref.: No)	2.56	1.18-5.54	0.016*

OR, Odds ratio; 95% CI, 95% confidence interval; Ref., Reference category. *P <0.05. Model 1: Odds ratio adjusted for factors from Model 1. Model 2: Odds ratio adjusted by factors from Models 1 and 2. Model 3: Odds ratio adjusted for factors from Models 1, 2, and 3.

## 4 Discussion

Our study showed that adherence to the MNT is associated with good glycemic control, regardless of socioeconomic factors. Among the factors that most interfered with this adherence was difficulty in maintaining the prescribed amount of food and subsequent mealtimes. We also found that physical activity, consolidated as one of the pillars of diabetes treatment, did not influence the glycemic control of our patients, although a few did so regularly. We also observed that in our highly admixed population, the percentage of ancestry was not an influencing factor for better glycemic control, suggesting that further studies using genomic ancestry should be conducted in other admixed populations to validate our findings.

Brazil has the third highest number of patients diagnosed with T1D (29), with only 13% of these patients having adequate glycemic control (30). Peres et al. (2022) also showed that patients with T1D have inadequate glycemic control in the richest and most developed regions of Brazil (20). Maranhão is a state in Northeast Brazil with one of the lowest Human Development Indexes (*Índice de Desenvolvimento Humano- IDH*) in the country (31), and a large part of the population lives in low socioeconomic conditions (32). In our evaluation implemented in a public service, we identified adequate glycemic control in 18.4% of the sample, demonstrating that although our patients were above the national average, most were not within the recommended HbA1c target. In the US, it has also been shown that the majority of children and adults with T1D were not meeting the HbA1c targets set by the American Diabetes Association (ADA) (21).

The literature indicates that along with good glycemic control and insulin replacement, the best way to reduce the chronic complications of diabetes is to educate the patient and their family (4). Guidance is required for proper MNT (one of the options is CHOC) and physical activity (7). Previous research has shown that low dietary adherence is related to poor glycemic control (10) (11). Similarly, The Diabetes Control and Complications Trial showed that poor adherence to a healthy diet was associated with poor glycemic control and increased insulin requirements in adults and youths with T1D (18). Corroborating the data above, in our hierarchical approach, we demonstrated that patients who had high dietary adherence was positively associated with good glycemic control, even after adjusting for sociodemographic conditions.

Adherence to the MNT prescribed for our patients with T1D was 39.5%, and when questioned, they cited the main difficulties encountered in adhering to the diet, maintaining the amount of prescribed food, and following meal times, similar to those found by Gomes et al. (33). Regarding the characteristics of the diet, it was also observed that the greater the difficulty in avoiding sugar and sweeteners, the poorer the HbA1c level.

Approximately 65% of our sample received personalized dietary advice, and only 16% performed CHOC. However, participants whose carbohydrates were counted had the highest percentage of adherence to the diet, although this was not statistically significant. A previous study showed the benefits of CHOC, leading to better glycemic control, but requiring constant monitoring of blood glucose levels. The same study included a sample of patients with T1D who had adequate family support and a high socioeconomic level, which was quite different from our sample (34). Meta-analyses have shown that HbA1c levels are significantly lower in patients with T1D who undergo CHOC (6, 7). However, this association was not observed in this study. We hypothesize that the low socioeconomic status of our population made it difficult to prescribe CHOC to our patients.

The Brazilian population consists of a heterogeneous mixture of Europeans, Africans, and Native Americans from different regions of Brazil (35). In a recently published study, we observed that in the Maranhão population, this miscegenation pattern differed from the average found in the rest of Brazil, with a lower percentage of European ancestry (46.5% vs. 68.1%) and a higher percentage of Africans and Native Americans (approximately 25% each versus 19.6% Africans and 11.6% American Indians). There was also a correlation between self-reported color and genomic ancestry, suggesting an adequate perception of color in our population (36). In the current study, we highlighted that despite being highly admixed, almost half of the patients with T1D from Maranhão had more than 60% European ancestry.

In several studies using self-reported colors, non-white T1D patients had worse glycemic control (22, 23), which was not observed in our study. When we used percentages of genomic ancestry, they showed no statistically significant accuracy in predicting good glycemic control, corroborating the findings of another large Brazilian study (33). We considered the possibility that the high degree of European ancestry and adequate self-perception of this miscegenation by our patients may have minimized the differences between racial minorities. New research in other admixed populations will be relevant to compare our results.

The direct effect of physical exercise on glycemic control in T1D remains controversial; however, there are significant benefits in reducing cardiovascular risk, controlling weight, and promoting well-being (37). In our study, we did not find a significant improvement in glycemic control in patients who practiced physical exercise. A 2016 study found that decompensated diabetes, fear of hypoglycemia, and other exercise-related fears were the main barriers to an active lifestyle in children and adolescents with T1D (38), which could explain why only 30% of our sample performed regular exercise. However, we observed that those who regularly performed physical activity were more adherent to their diet, indicating a possible synergistic effect. In another study of the same population, we observed that physical activity was related to a better quality of life in these patients (39), demonstrating that exercise positively influences treatment, even if it does not directly improve control. In addition, evidence has suggested that neuroticism and other personality traits are associated with adopting a physical exercise routine (14). Therefore, future studies could incorporate personality analysis to explore the linkage between lifestyle behaviors and glycemic control in the T1D population.

Adolescence is a phase marked by major physical and emotional changes that negatively affect diabetes control. Adolescents with T1D tend to be less vigilant with blood glucose monitoring and insulin application, which can make it difficult to control HbA1c (38, 40). Our research showed that age > 40 years was associated with better glycemic control compared to children and adolescents, confirming the results of the aforementioned studies. Although treatment is a major challenge at different stages of life, especially during adolescence, interdisciplinary care and family support can improve care management (40).

Surprisingly, men were associated with better glycemic control in this study, differing from other studies where there was no difference between sexes (25, 41) and from European multicenter research where female sex was associated with better control (42). We believe that the small number of participants in our study made this measurement difficult.

Our findings suggest that consistent adherence to dietary regimens is associated with good glycemic control after adjusting for sociodemographic and genomic ancestry factors in an admixed population of Northeast Brazil of T1D patients. Identifying possible barriers to adherence to the proposed type of MNT will improve patient engagement with nonpharmacological treatment of diabetes. Although exercise has not been related to improved control, its benefits are an important in the management of T1D. We also observed that in our highly admixed population, the percentage of ancestry and self-reported color/race were not an influencing factor for better glycemic control, suggesting that further studies using genomic ancestry should be conducted in other admixed populations to validate our findings. Therefore, future studies with longitudinal follow-up should investigate the effectiveness of specific dietary protocols and different physical activity routines on glycemic control in people with T1D.

## Data availability statement

The raw data supporting the conclusions of this article will be made available by the authors, without undue reservation.

## Ethics statement

The studies involving humans were approved by This research was approved by the ethics committee from the University Hospital of the Federal University of Maranhão (HUUFMA) under opinion number 59795116.9.0000.5086. The studies were conducted in accordance with the local legislation and institutional requirements. Written informed consent for participation in this study was provided by the participants’ legal guardians/next of kin.

## Author contributions

RA, MG, MF: Conceptualization, supervision, project administration, investigation, writing original draft, review, and editing. VaR: Methodology, statistical formal analysis, visualization, writing original draft, review and editing. DS: Methodology, review, investigation, visualization. AS: samples management and data collection. JDMFA, AA, DL, MT, GN, ViR, MM, AF, CN, CP: data collection, review and editing. All authors contributed to the article and approved the submitted version.
